# Targeting Ras with protein engineering

**DOI:** 10.18632/oncotarget.28469

**Published:** 2023-07-01

**Authors:** Atilio Tomazini, Julia M. Shifman

**Affiliations:** ^1^Department of Biological Chemistry, The Alexander Silberman Institute of Life Sciences, The Hebrew University of Jerusalem, Jerusalem 9190401, Israel

**Keywords:** Ras oncogene, anti-Ras therapeutics, Ras targeting, protein engineering, protein design

## Abstract

Ras proteins are small GTPases that regulate cell growth and division. Mutations in Ras genes are associated with many types of cancer, making them attractive targets for cancer therapy. Despite extensive efforts, targeting Ras proteins with small molecules has been extremely challenging due to Ras’s mostly flat surface and lack of small molecule-binding cavities. These challenges were recently overcome by the development of the first covalent small-molecule anti-Ras drug, sotorasib, highlighting the efficacy of Ras inhibition as a therapeutic strategy. However, this drug exclusively inhibits the Ras G12C mutant, which is not a prevalent mutation in most cancer types. Unlike the G12C variant, other Ras oncogenic mutants lack reactive cysteines, rendering them unsuitable for targeting via the same strategy. Protein engineering has emerged as a promising method to target Ras, as engineered proteins have the ability to recognize various surfaces with high affinity and specificity. Over the past few years, scientists have engineered antibodies, natural Ras effectors, and novel binding domains to bind to Ras and counteract its carcinogenic activities via a variety of strategies. These include inhibiting Ras-effector interactions, disrupting Ras dimerization, interrupting Ras nucleotide exchange, stimulating Ras interaction with tumor suppressor genes, and promoting Ras degradation. In parallel, significant advancements have been made in intracellular protein delivery, enabling the delivery of the engineered anti-Ras agents into the cellular cytoplasm. These advances offer a promising path for targeting Ras proteins and other challenging drug targets, opening up new opportunities for drug discovery and development.

## INTRODUCTION TO RAS

**Figure 1:**
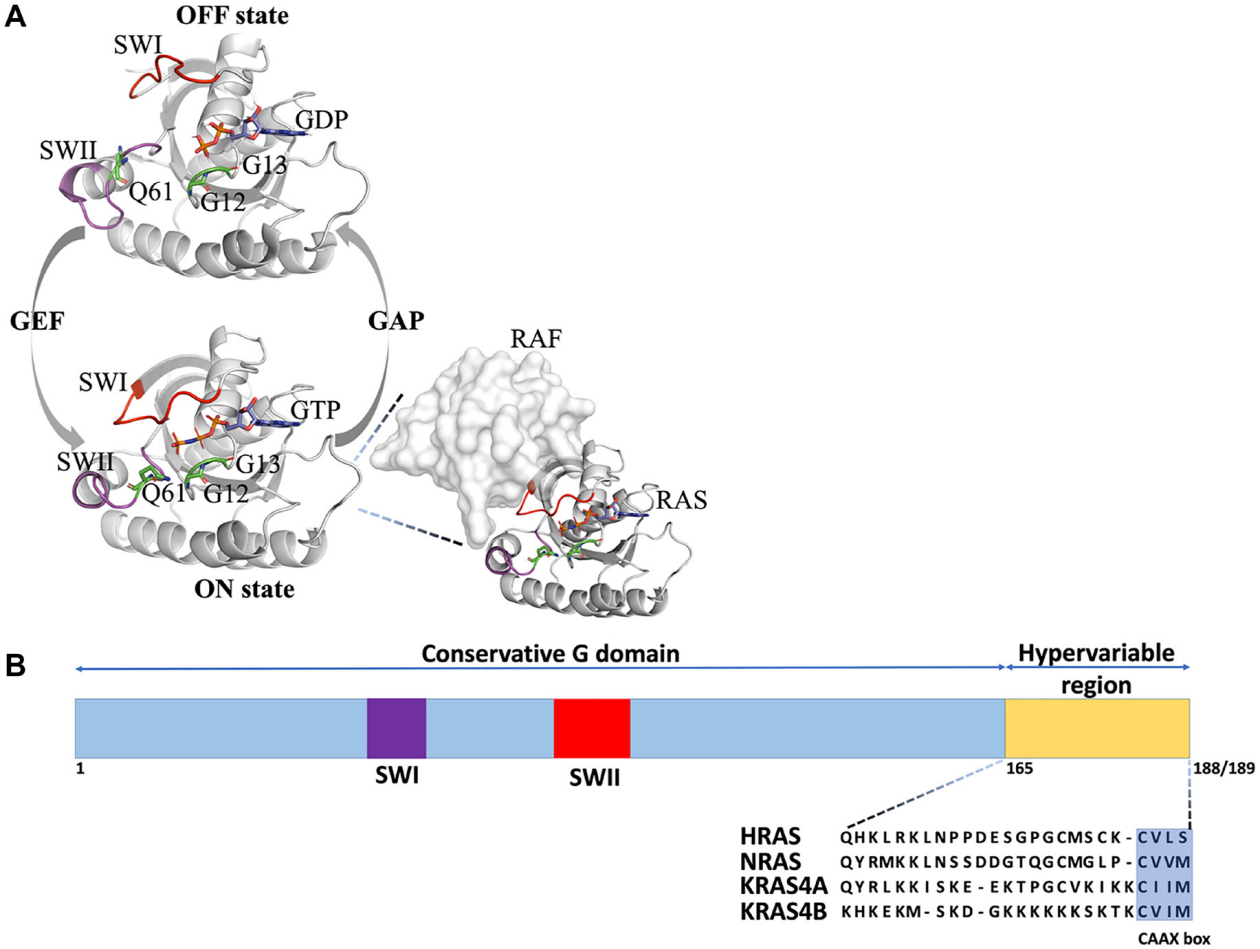
2D and 3D structure of Ras isoforms. (**A**). The Ras GDP/GTP switching cycle. Ras is activated upon GTP binding aided by binding to Guanine nucleotide Exchange Factors (GEFs), which puts Ras proteins into the ON state (SWI close to SWII). In this state, it can bind to effectors such as Ras (shown in the insert on the right) and activate downstream signaling pathways. When GTP is hydrolyzed to GDP, the effectors are released, switching RAS into the OFF state (SWI far from SWII and both switches are mobile). Ras activity is accelerated by binding to GTPase Activating Proteins (GAPs). Positions of the most frequent oncogenic Ras mutations (G12, G13, and Q61) are shown as green sticks and GTP is shown as sticks and colored by atom type, (**B**) Schematic representation of the Ras gene structure. The G domains including switches I and II (SWI and SWII, respectively) (amino acids 1–165) of HRAS (UniProtKB entry: P01112), NRAS (UniProtKB entry: P01111), KRAS_A (UniProtKB entry: P01116) and KRAS_B (UniProtKB entry: P01116-2) are highly conserved (90–100% identical). The hypervariable region (HVR) (amino acids 165–189) comprises the carboxy-terminal CAAX box motif.

Ras is a master regulator of many processes in the cell including cell cycle progression, cell migration, adhesion, differentiation, and apoptosis [1, 2]. Ras functions by cycling between an active, GTP-bound state (Ras-GTP), and an inactive, GDP-bound state (Ras-GDP) [3, 4]. Switching to the active state of Ras is stimulated by binding of a guanine nucleotide exchange factor (GEF) that increases the nucleotide dissociation rate of Ras and promotes loading with GTP, which is present in excess over GDP in the intracellular environment. Switching to the inactive Ras state occurs upon GTP hydrolysis to GDP. This process is increased by several orders of magnitude on binding of a GTPase-activating proteins (GAPs) that stimulate the intrinsically low GTPase activity of Ras [5]. In the active GTP-bound form, Ras is able to bind and activate its multiple effector proteins (Figure 1A). Once GTP is hydrolyzed, the affinity of Ras to effectors is substantially reduced, breaking its interactions with downstream targets.

**Figure 2:**
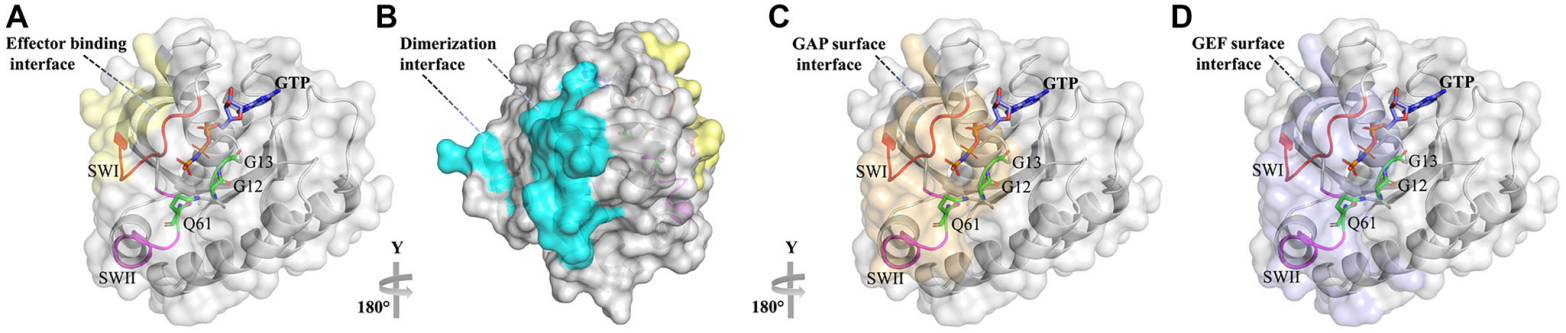
Ras with its various binding interfaces. Ras is shown in the surface representation with the GTP molecule colored by atom type. (**A**) Effector binding interface is colored in yellow. (**B**) Ras dimerization interface is colored in cyan. (**C**) GAP binding interface is colored in beige. (**D**) GEF binding interface is colored in violet. The interfaces are colored by analyzing the structures of Ras in complex with Raf (PDB id:1C1Y), GAP (PDB id: 1WQ1), and GEF (PDB id: 6D5W) and according to the Ras dimer contacted area described in ref [20].

Three Ras genes are found in humans resulting in four Ras isoform proteins after splicing (HRAS, NRAS, and KRAS4A and KRAS4B) that show differential expression in different tissues [6–10]. These Ras isoforms share a conserved G-domain (residues 5-164) that performs GTP hydrolysis and a highly variable C-terminal domain (residues 165-188/189) that targets Ras to the membrane (Figure 1B) [11]. Targeting to the membrane is achieved through posttranslational modifications that are sequence-specific, likely explaining non-redundant biological functions and mutational spectra of the Ras isoforms in human cancers [12, 13]. The G-domain contains a conserved nucleotide-binding pocket (residues 32-40) and the so-called switch regions, switch I (residues 32-38), which constitutes the effector binding interface and switch II that is close to the effector binding site (residues 59-67) (Figure 1A and 2A) [10, 14]. Both switches are mobile and change their conformation depending on the nature of the nucleotide-bound state (GDP or GTP) [11]. Opposite to the effector binding interface (Figure 2A), the suggested Ras dimerization interface is located that includes helixes α4 and α5 and a loop between β2 and β3 [15] (Figure 2B). Some studies show that dimerization of Ras on the membrane is stimulated by the C-terminal hypervariable domain and is important for Ras nanoclustering and downstream signaling [16–19]. GAP and GEF bind to the RAS site overlapping with the effector binding region, covering the nucleotide and interacting mainly with the switch I and II regions (Figure 2C and 2D).

More than a hundred of putative Ras effectors have been identified, which all contain an 80-100-amino-acid RAS-Association (RA) domain or RAS Binding Domain (RBD) that exhibits the ubiquitin fold [20]. All the effectors bind to the switch I site on Ras, forming an antiparallel intermolecular β-sheet between β2 of the effector RBD domain and β2 of Ras and creating a network of hydrogen bonds across the binding interface (Figure 1A) [21, 22]. Ras binds to effectors with high affinity when bound to GTP; GTP conversion to GDP results in conformational changes in switches I and II and increases their mobility, significantly reducing Ras binding affinity to all its effectors [23]. The *in vitro* binding affinity of Ras-GTP to effectors ranges from 10^−8^–10^−4^ M and differs significantly depending on the effector, thus establishing the hierarchy of various Ras-controlled processes [24].

The most well-studied Ras effectors belong to Raf, RalGDS, and PI3Ks families which all stimulate pro-cancer pathways. Raf proteins are Ser/Thr protein kinases that activate the MAPK/ERK pathway resulting in cell proliferation and differentiation [25]. Ral guanine nucleotide dissociation stimulator (RalGDS) activates small GTPases RalA and RalB, turning on pathways that mediate cell transformation and cytoskeletal reorganization [26–28]. PI3Ks are lipid kinases that by phosphorylating phosphoinositides activate Akt family Ser/Thr kinases, which, in turn, are pivotal in the inhibition of apoptosis and the promotion of cell survival, growth, and migration [29]. The other group of Ras effectors belongs to the RASSF family and are known cancer suppressor genes. Among RASSF effectors, RASSF5 (or Nore1A) binds to Ras with the highest affinity and stimulates pro-apoptotic and pro-senescence pathways [30]. This protein is inactivated by promoter hypermethylation in numerous cancer cell lines and primary cancers [31, 32].

Several point mutations in Ras are associated with cancer. Roughly 19 to 30% of human tumors contain Ras mutations with the highest frequency occurring in pancreatic (98%), colorectal (52%), and lung carcinomas (32%) [24, 33–35]. 98% of all oncogenic mutations are located at three sites on Ras: 12, 13, and 61, all situated near the nucleotide binding site (Figure 1A) [36]. 70% of patients with Ras mutations have one of five allele variants (G12D, G12V, G12C, G13D, and Q61R) [35]. Interestingly, the frequency of oncogenic mutations differs depending on the Ras isotype. While G12 is the most frequent site of mutations in KRAS, especially in pancreatic and lung adenocarcinoma, Q61 is most commonly mutated in HRAS and NRAS and is frequently found in bladder urothelial, papillary thyroid carcinoma, skin cutaneous melanoma and acute myeloid leukemia [35]. Oncogenic mutations increase the concentration of the GTP-bound active Ras in cells, as such mutants are not able to convert GTP to GDP, thus locking Ras in the “on” state where it constantly activates cell cycle progression and division [8–11]. Breaking down of the Ras signaling is due to two main mechanisms: decrease in intrinsic catalytic activity of a Ras mutant and its decreased affinity for GAPs, which stimulate the intrinsic GTPase activity. In addition, oncogenic mutations change binding affinity of Ras to various effectors, perturbing the Ras signaling networks [24].

While Ras’s relationship to cancer has been long established [37], it has been considered an undruggable target for many years. The main difficulty in targeting Ras with small molecules comes from the apparent lack of well-defined pockets on the molecular surface of Ras where such molecules could bind [38]. The only cavity in Ras is the nucleotide-binding pocket itself; however, this pocket exhibits an intrinsically high affinity for GDP and GTP, making this site problematic for drug design efforts [39]. Another potential inhibition site is the conserved effector binding interface. This interface, however, is flat and lacks any cavities (Figure 2A) [39], providing no surface for a small molecule to bind. In spite of these difficulties, in recent years we witnessed a resurgence of studies reporting Ras-directed inhibitors including small molecules, peptidomimetics, and proteins [40]. Recent structural studies and molecular dynamics simulations identified two new potential pockets on Ras that can be drugged: a pocket close to the switch II region, which is only present in the inactive GDP-bound Ras conformation, and a pocket between the two switches in both the active and the inactive Ras states [41–43].

The first pocket has been utilized to obtain several small molecule inhibitors that covalently attach to a cysteine in the Ras G12C mutant (ARS-1620, AMG-510, ARS-853, and MRTX 849) [44–47] and culminated in the development of two anti-Ras drugs that were granted accelerated FDA approval for treatment of K-Ras G12C-mutated non-small cell lung cancers: Sotorasib (AMG 510, Lumakras^™^, Amgen, Inc.) and Adagrasib (MRTX849, KRAZATI^™^, Mirati Therapeutics) [48–50]. Moreover, this pocket has been utilized to obtain MRTX1133, a small noncovalent inhibitor of K-Ras G12D, that is now being explored in clinical trials [51, 52]. The druggability of the second pocket was demonstrated by several small-molecule ligands that led to inhibition of downstream signaling in K-Ras and H-Ras mutant cells [53–56]. These recent successes of small-molecule Ras inhibitors broke the paradigm of Ras being undruggable and revealed the high potential of targeting Ras in various cancer types. The possibilities for anti-Ras drug design expand significantly if we do not limit ourselves to small molecule inhibitors. The interaction sites of Ras with its effectors, with GAP and GEF, and the Ras dimerization interface could be all targeted with protein-based inhibitors, providing various strategies for therapeutic intervention. While the development of small-molecule Ras inhibitors has been reviewed elsewhere [40], we focus our review on protein-based Ras inhibitors, describing the methods for their engineering, various scaffolds used for inhibitor design, and prospects for delivery of the designed Ras inhibitors into the cellular cytoplasm, where Ras is located.

## DIFFERENT SCAFFOLDS FOR PROTEIN ENGINEERING

Unlike small molecule drug candidates, protein domains do not require a cavity to bind to their targets. They often interact through large surface areas and hence possess the ability to bind their targets with extremely high affinity and specificity [57]. In fact, engineered proteins unlike small molecules frequently exhibit monospecificity for their targets *in vivo* in spite of the presence of many highly similar proteins in the cell [58, 59]. Three classes of protein scaffolds could be utilized for engineering of protein-based inhibitors including antibodies in various formats, natural protein effectors, and novel binding domains that are not related to the target protein [60]. The most widely used scaffolds for protein engineering are antibodies that possess the natural ability to hypermutate within the six Complementary Determining Regions (CDRs) and to acquire high affinity to any target protein [61]. Antibodies have been engineered for high-affinity binding to various drug targets by the pharmaceutical industry with a number of antibodies already approved as drugs and many more being explored in clinical trials [62].

Nevertheless, antibodies have several well-known limitations, including the high cost of recombinant production [63], potential undesired effector functions [64, 65], suboptimal tissue penetration, and the considerable intellectual property barriers to their development [66]. Some of these disadvantages could be overcome by converting full-length antibodies into their smaller versions such as fragment antigen-biding (Fabs), variable domains (Fv) or single chain variable domains (scFv) [67]. However, antibodies or their fragments are not unique in their ability to recognize various target proteins. Alternative protein scaffolds of the non-antibody format have been widely used in various protein engineering studies with the goal of obtaining high-affinity binders for various targets.

Natural protein effectors are attractive scaffolds for inhibitor design as they already bind to the target protein with initial affinity and interact with the desired epitope. A few mutations in such natural effectors could be engineered to enhance binding affinity and their binding specificity, converting them into powerful inhibitors that are non-toxic and are likely non-immunogenic due to their endogenous nature [60]. For example, ubiquitin has been engineered to modulate various enzymes in the ubiquitin pathway [59], natural broad tissue inhibitors of metalloproteases (TIMPs) have been converted into highly specific inhibitors [68, 69], and angiotensin-converting enzyme 2 (ACE2) dimers have been engineered to inhibit the interaction between the receptor binding domain of SARS-CoV2 and ACE2 [70].

In addition to natural effectors, different novel binding domains have been utilized to target various proteins in diseases. Such novel binding domains (also called alternative scaffolds) usually possess high structural robustness, high solubility, and high expression yield - characteristics that make them attractive candidates for drug design. These novel binding domains do not have any biological relationship to the target protein and could be evolved to exhibit high geometrical and physico-chemical complementarity to the target, resulting in nM to pM binders. The most prominent scaffolds include monobodies, affibodies, DARPins, adnectins, ANTICALIN^®^, and knottins [71–73].

**Figure 3:**
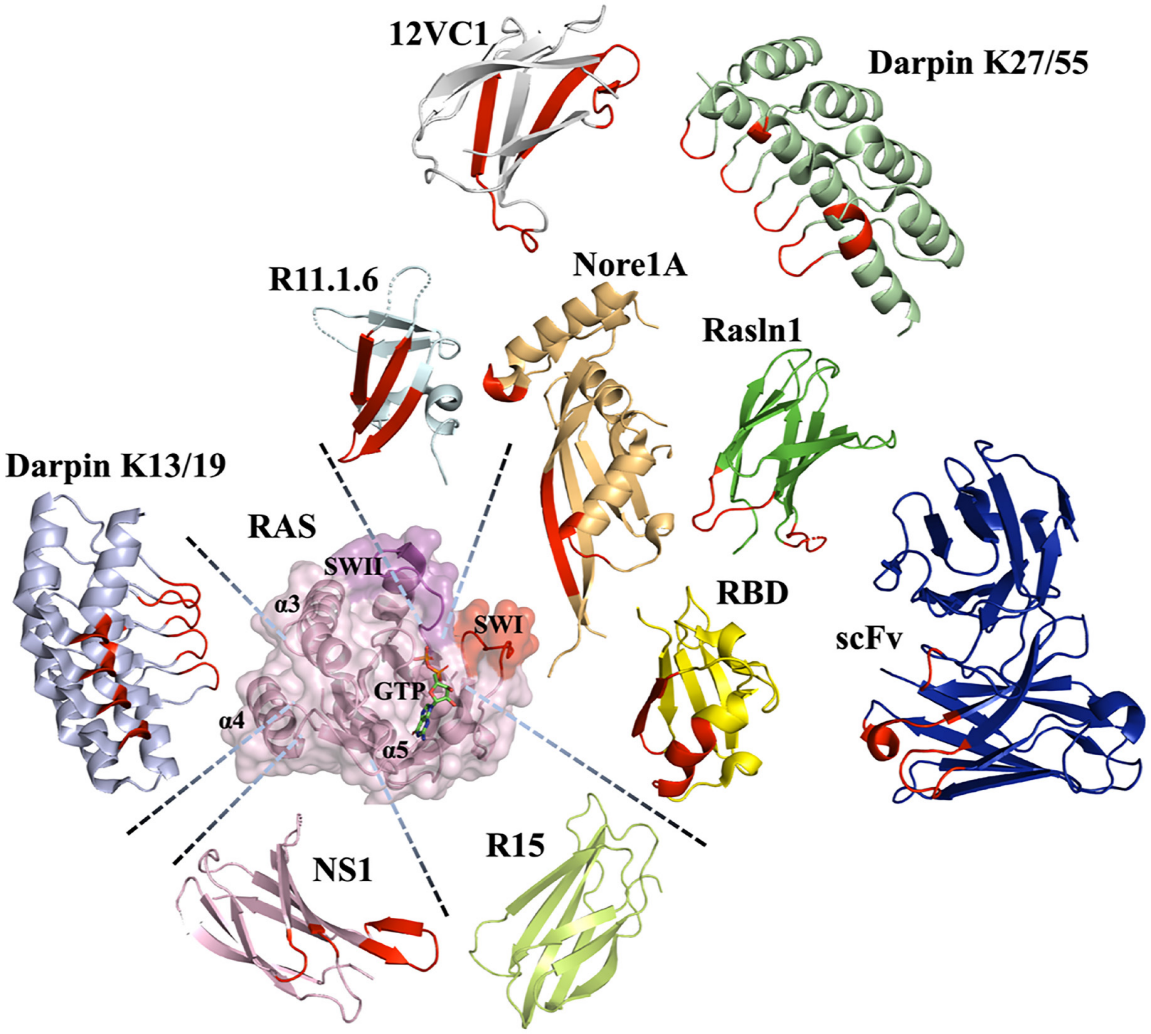
Various scaffolds utilized to engineer binders to Ras and their binding epitopes. The H-RAS structure (PDB id: 5e95) (light pink) with Switch (SW) I (red) and II (magenta), dimer contact (α4-α5) regions and GTP stick chain are indicated. Scaffolds described to targeting Ras showed respecting the contact position with Red highlight the contact area: R11.1.6 (PDB id: 5UFQ) (Pale cyan color), Nore1A (RASSF5) (PDB id: 3DDC) (Wheat color), 12VC1 (PDB id: 7L0F) (light gray color), Rasln 1 monobody, expected position (PDB id: 6XAY used as folding reference) (Lemon color), scFv (PDB id: 2UZI) (Blue color), RBD (PDB id: 4G0N) (Yellow color), DARPin k27 (PDB id: 5O2S) (Palegreen color), NS1 (synthetic binding protein (monobody) based on fibronectin type III) (PDB id: 5E95) (Light Pink color), DARPin k13 (PDB id: 6H46) (Light Blue color) and R15 (predicted model by AlphaFold) (Lemon color).

Interestingly, all classes of protein scaffolds, including antibodies, natural effectors, and novel binding domains, have been utilized for engineering of Ras binders, allowing scientists to target various sites on the Ras surface and to explore different strategies for inhibiting Ras oncogenesis (Figure 3) [39, 74–76].

## PROTEIN ENGINEERING METHODOLOGIES

Protein inhibitors could be engineered through experimental directed evolution approaches, through computational design, or through a combination of the two methodologies. Experimental techniques for protein engineering include different display technologies such as phage display (PD) [77], yeast surface display (YSD) [78], ribosome display [79], or mRNA display [80]. In addition to these display approaches, an intracellular antibody capture technique (IAC) has been developed to isolate high-affinity antibodies in the form of single VH domains that could function in the reducing environment of eukaryotic cells [81]. IAC, based on the yeast two-hybrid approach, results in growth of the yeast cells only upon interaction between the target and the antibody binder. In all protein engineering experiments, large combinatorial libraries of protein mutants are first designed and constructed. These libraries are then expressed and the target protein is used as a ‘bait’ to select for binders. After several rounds of selection, the DNA of the selected binders is recovered and sequenced. While early studies sequenced one mutant at a time, more recent studies utilized a deep sequencing approach that allows us to obtain a sequence profile of mutants compatible with high-affinity binding, revealing the importance of each residue for binding affinity [82].

Protein binders could be also engineered to possess high specificity by addition of negative selection steps, i.e., selection experiments where binders to unwanted targets are eliminated from the selection pool. Different binder display approaches have been equally successful in obtaining high-affinity binders, although a certain approach might be more suited for a particular scaffold protein. All the display technologies, however, are limited by the number of protein mutants that they could assay. As such, IAC can explore ~10^6^ variants, YSD - 10^7^-10^8^ mutants, PD - up to 10^10^, and ribosome and mRNA display - up to 10^15^. While these numbers might seem large, they imply that only 5–11 positions in a protein could be fully randomized to all twenty amino acids. This number is considerably smaller than the number of positions in an average protein-protein binding interface [83], meaning that for an efficient binder engineering experiment to work, randomization should be restricted to a smaller subset of amino acids, using a focused rather than a fully random library. Focusing of combinatorial libraries is quite common and could be easily performed using computational techniques or by restricting amino acid choices to only a few that are observed most commonly in protein-protein interfaces [84, 85].

When engineering protein binders from antibodies and novel binding domains, one must consider that such binders could target various epitopes on the target protein and would not be necessarily inhibitory. In some cases, such binders could be even activating. Thus, a binding epitope on the target has to be established using either structural studies or competition assays using another binder with the known epitope. Unlike antibodies and novel protein domains, natural effectors bind to a predetermined “natural” epitope on the target protein; this epitope does not change upon affinity maturation, thus giving natural effectors an advantage over other scaffolds during inhibitor design [59].

In addition to directed evolution approaches, several computational methods for protein binder design have been proposed [86–93]. Unlike experimentally selected binders, computationally designed binders could be rationally designed to recognize any desired binding epitope on the target protein. However, purely computationally designed binders frequently exhibited weak starting binding affinities and needed additional affinity maturation using experimental protein engineering approaches such as YSD to achieve nM and pM affinities. While de novo binder design remains challenging, computational methods proved to be extremely successful in designing focused libraries of binders for YSD experiments. In such an approach, relatively small libraries of protein mutants could be designed based on computational binding affinity predictions, narrowing down the number of choices to only the most promising ones and allowing to assay all of them with directed evolution [57, 94]. Thus, through a combination of computational and experimental approaches, protein inhibitors with superior affinity and specificity have been discovered [57, 69, 95, 96].

## ENGINEERING PROTEIN INHIBITORS OF RAS ONCOGENIC SIGNALING

Ras activity involves a number of protein-protein interactions that are conveyed through several binding interfaces including Ras-effector binding interface, Ras dimerization interface, and Ras interaction sites with GAP and GEF (Figure 2). Interference with any of these interactions could in principle lead to the inhibition of Ras oncogenic signaling, hence all these interfaces have been targeted with protein binders.

The first engineered anti-Ras protein inhibitor was a single-chain variable fragment (scFv) that, when expressed intracellularly, colocalized with Ras at the plasma membrane and inhibited Ras activity in mammalian cells and tumor xenografts [97–99]. In further work, single variable domains termed iDabs (intracellular single variable domain antibody) [100, 101] were developed and optimized for solubility, stability, and specificity. Using an intracellular antibody capture (IAC) approach with H-Ras G12V as a bait, iDAbs were engineered by fully randomizing CDR loops on the iDAb scaffold. The best variant, iDab#6, bound specifically to the activated GTP-bound form of Ras with low nM affinity and exhibited substantially weaker affinity to Ras-GDP. Crystal structure of the corresponding single chain scFv (Figure 3) in complex with Ras was solved and showed iDab#6 interacting with switches I and II on Ras, resulting in competitive inhibition of Ras-effector interactions. iDab#6 was used to investigate the involvement of mutant Ras-dependent signaling pathways at the beginning of cancer [101] and its long-term progression [102]. This protein prevented tumor initiation and controlled cancer cell development in a transgenic model of lung cancer, but did not result in tumor regression [102]. These data first demonstrated that mutant Ras–effector interactions are necessary for cancer progression and that inhibiting Ras–effector interactions is a valid strategy for cancer therapy although inhibition of additional downstream targets might be necessary at the later stages of cancer. iDAb#6 was further utilized to screen for small-molecule inhibitors targeting the same effector-binding interface on Ras. A number of such small molecules were isolated and the initial leads were optimized based on structural analysis of Ras/inhibitor complexes, resulting in compounds that could penetrate cells, prevent Ras-effector interactions, and inhibit endogenous Ras signaling [103].

Ras effectors are natural starting points for disrupting Ras/effector interactions as such proteins already bind to the correct binding epitope. Evolving them to enhance their affinity to Ras, would produce molecules that compete with Ras effectors and block all downstream signaling [21]. In such a strategy, Raf RBD was randomized at 14 positions that contact switch I on Ras with the library biased to retain WT identity. Phage display selection for binding to H-Ras-GTP yielded several RBD variants with low nM affinity to Ras [39]. X-ray structures of H-Ras G12V in complex with RBDv1 and RBDv12 revealed that affinity-matured proteins retain the binding mode of WT Raf RBD and improve affinity through a number of new intermolecular interactions with the switch I on Ras (Figure 3). When expressed intracellularly in cancer cell lines, these variants exhibited high specificity toward the mutated Ras, resulting in a reduction in Ras signaling, inhibition of cell growth, and induction of apoptosis. When tested in organoids of patient-derived colorectal cancer models with Ras mutations, the RBD variants inhibited cancer progression in only a subset of cases, revealing that not all Ras-mutated tumors depended on Ras signaling for proliferation.

Another Ras-effector-based inhibitor was engineered using the RBD of Nore1A (Figure 3) also referred to as Ras Association Domain Family 5 (RASSF5) [94]. Nore1A is a known tumor suppressor gene, expression of which is frequently downregulated in cancer cells [31, 32]. While catalytically inactive, Nore1A serves as an adaptor protein that links Ras signaling to pro-apoptotic and pro-senescence pathways [30]. In a study by Singh et al, computational design was used to optimize intermolecular interactions of Nore1A with Ras; subsequently small combinatorial library of Nore1A mutants was constructed based on computational results and YSD was utilized to select high-affinity binders to Ras-GTP and/or Ras-GDP [94]. Unlike other previously engineered Ras binders, the engineered Nore1A variants when expressed in A549 lung adenocarcinoma cells, not only inhibited Ras-regulated pro-cancer pathways but also promoted anticancer pathways stimulated by Nore1A. The results of this study thus proved that strengthening interaction between Ras and a tumor suppressor protein could be an attractive strategy for therapeutic intervention.

In another study, Ras binder was engineered based on a fibronectin type III domain (FN3), also referred to as monobody, ~100-amino-acid domain that lacks disulfide bonds and exhibits immunoglobulin fold that is common to all antibody fragments [104, 105]. The two flexible loops BC and FG of this scaffold correspond to antibody CDRH1 and CDRH3 loops, respectively [76], and could be evolved for interaction with target proteins [106]. Allowing mutations to occur in additional regions of this domain proved advantageous in selecting high-affinity binders for some targets. Randomization of monobody residues on β-strands C and D and the FG and CD loops and subsequent selection for binding to H-Ras with phage display yielded several high-affinity Ras binders [74]. Among them, NS1 did not discriminate between the GTP- and GDP- states of Ras and bound to H-Ras and K-Ras but not to N-Ras with ~10^−8^ M affinity. X-ray structure of the NS1 in complex with Ras-GDP showed that NS1 interacts with the Ras helixes α4 and α5 that constitute the Ras dimerization interface (Figure 2B and Figure 3), which is important for Ras nanocluster formation on the membrane and subsequent signaling in the cell. Disruption of Ras dimerization by NS1 inhibited oncogenic H- and K-Ras signaling and transformation, leading to inhibition of C-RAF/B-RAF heteromerization and activation. Further study demonstrated that NS1 potently inhibits cell growth in 3D cultures of K-Ras mutated cells and blocks the oncogenic K-Ras-driven tumor growth *in vivo*, providing proof of concept for targeting the α4–α5 dimerization interface as an approach to inhibit Ras-driven tumorigenesis [107]. An additional Ras binder from a monobody scaffold was evolved to bind to the nucleotide-free state of Ras. This R15 monobody bound with nM affinity to all three Ras isoforms and inhibited nucleotide exchange in fast-exchanging mutants. Furthermore, its intracellular expression resulted in reduced tumor-forming capacity of patient-derived xenographs [108].

Another study also used a monobody scaffold for Ras binder engineering. In this study the FG loop on a monobody was randomized, while the BC loop was grafted from iDAb#6 described above [101] and mRNA display followed by in-cell screening was applied to identify a binder to a H-Ras G12V mutant [76]. The initial binder called RasIn1 (Ras Intrabody 1) (Figure 3) exhibited μM affinity to H-Ras G12V (GTP), showing high preference for the Ras GTP-bound state. Further affinity maturation resulted in RasIn2 with a K_D_ of 120 nM. Mutagenesis and biochemical studies demonstrated that RasIns recognize switch I region on Ras and are able to compete with Raf RBD. Furthermore, they colocalized with H-Ras and K-Ras when expressed intracellularly and showed high specificity against other Ras homologues.

Many recent studies explore the selective degradation of disease-associated proteins as a therapeutic strategy [109]. In such studies, Proteolysis-targeting chimeras (PROTACs) are constructed that constitute a fusion between a protein of interest binding module and E3 ubiquitin ligase that ubiquitinates and subsequently degrades the protein. To test this approach on Ras, another monobody was engineered to bind with high selectivity to the active GTP-bound state of two oncogenic RAS mutants, K-RAS G12V and K-RAS G12C as compared to wild-type Ras [110, 111]. The best-engineered protein, 12VC1 (Figure 3), showed 400-fold better affinity for K-RAS G12C over the wild-type protein and formed interactions with switches I and II on Ras as well as with the bound nucleotide. Subsequently, 12VC1 was fused with E3 ubiquitin ligase subunit VHL that sends interacting proteins to degradation through the ubiquitin pathway [112]. Indeed, expression of 12VC1-VHL in cells resulted in selective degradation of Ras mutants, producing a more powerful effect compared to simple Ras activity inhibition. A strategy of Ras degradation has been also utilized in a recent study, where a Ras/RAP1 specific endopeptidase, which cleaves Ras between residues Y32 and D33, reduced tumor burden in three mouse xenografts [113]. These results reveal that selective degradation of Ras mutants with chimera proteins that recognize Ras is another promising strategy against Ras-driven cancers. These results reveal that selective degradation of Ras mutants with chimera proteins that recognize Ras might be another promising strategy against Ras-driven cancers.

Additional scaffolds that have been used for Ras inhibitor design are DARPins, small proteins, based on an ankyrin repeat protein, that possess a concave surface with four loops, all of which could be randomized in binding selection experiments [114]. High stability, lack of disulfide bonds, and high expression yield make DARPin an attractive scaffold for therapeutic protein engineering with several DARPins entering clinical trials for other targets [115]. Phage display technology was used to isolate two variant DARPins, one that targets the GAP binding interface on K-Ras and inhibits nucleotide exchange (K27) and one that targets the effector bindings interface (K55) (Figure 3) [116]. K27 bound with low nM affinity to the GDP-form of K-Ras G12V and 1000-times weaker to the GTP form of Ras. K55, on the other hand, showed stronger binding to Ras-GTP (K_D_ = 167 nM) and no detectible binding to Ras-GDP. Both proteins were not selective for any of the Ras oncogenic mutations. Crystal structures of both DARPins in complex with Ras have been solved, revealing the key interactions (Figure 3). Intracellular expression of K27 in human colorectal carcinoma HCT116 cell line resulted in a significant reduction of Ras signaling, in particularly reduction in the level of phosphorylated ERK, and slowed cellular growth. Thus, K27 demonstrates the feasibility of nucleotide exchange inhibition in Ras as a therapeutic strategy. Additional DARPins have been engineered to target an allosteric site that includes α3/loop 7/α4 and is specific to K-Ras isotype [117]. DARPins directed to this site (K13 and K19) showed nucleotide independent binding, resulted in inhibition of effector binding to K-Ras in cell-based essays, and impeded nucleotide exchange and Ras dimerization.

Another scaffold utilized for targeting Ras is the Sso7d protein from hyperthermophilic bacteria. This scaffold is small (7 kDa), possesses high thermostability (Tm of 98°C), and contains no cysteines and glycosylation sites, thus being perfectly suited for intracellular targeting [118]. Sso7d has been engineered by YSD to target specifically the K-Ras G12D mutant. Crystal structure of the engineered protein R11.1.6 in complex with Ras showed that the protein is bound to the switch II region of Ras, exhibiting an overlapping binding interface with Ras effectors (Figure 3). Binding measurements revealed that the protein recognizes equally well the GTP-bound state of all three Ras isotypes and several Ras oncogenic mutants. This protein was shown to directly compete with Raf and to reduce signaling through the Raf/MEK/ERK pathway [118]. However, it failed to inhibit signaling through both the MAPK and PI3K pathways in a panel of human cancer cell lines [119]. This absence of the observed effect in cells was explained by a mathematical model that predicted that only 16% of the K-Ras/Raf complexes were inhibited by the engineered protein in cellular conditions [119].

We have summarized all the described engineered Ras protein-based binders and their properties in Table 1.

**Table 1:** Engineered protein inhibitors of Ras

Scaffold	Name	Ras interaction site	Specificity	Kd, nM^*^	References
IgG	iDab#6	Switch I	Ras-GTP	2.6–10	[101]
monobody	NS1	α4–α5	Ras-GTP and Ras-GDP H- and K-Ras	13–67	[74]
monobody	Rasln-1 and -2	Switch I	Ras-GTP	2100; 120	[76]
monobody	12VC1	Switches I/II	K-Ras(G12C/V)-GTP	24–100	[110, 111]
monobody	R15	Nucleotide site	Apo-Ras	36	[108]
IgG	RT11	Switch I	Ras-GTP	4–17	[120]
monobody	JAM20	Switches I/II	Ras-GTP and Ras-GDP	5–30	[121]
Nore1A	T1	Switches I/II	Ras-GTP	560	[94]
RAF RBD	RBDv1 and RBDv12	Switch I	Ras-GTP	2.5–3.4	[39]
DARPin	K13 and K19	α3-α4	K-Ras-GTP and K-Ras-GDP	30; 10	[117]
DARPin	K27	Switch I	Ras-GDP	4	[116]
DARPin	K55	Switch I	Ras-GTP	167	[116]
Sso7d	R11.1.6	Switch II	Ras-GTP(G12D) and Ras-GDP	2–50	[118]
Ras Protease	RRSP	Switch I	Ras-GTP and Ras-GDP	−	[113]

^*^ *in vitro* binding affinity measured by SPR. A range is given when affinities to several Ras mutants and/or states are measured.

## PROSPECTS FOR INTRACELLULAR DELIVERY OF RAS BINDERS

Intracellular expression of these proteins in many cases results in a decrease in pro-cancer pathways and an increase in anti-cancer activities. However, the full therapeutic potential of such molecules cannot be readily realized without delivering these proteins into the cellular cytoplasm, where Ras is located. Since most proteins do not cross the cellular membrane on their own, a separate strategy should be devised for delivering Ras binders to the cytoplasm. In recent years, several new strategies for protein intracellular delivery have been reported. In one such an approach, scientists have been utilizing short, cell-penetrating peptides (CPPs) that could be fused to the desired cargo protein. Hundreds of highly positively charged, hydrophobic, and amphipathic CPPs of natural and synthetic origin have been identified over the years (summarized in the CPP database: https://webs.iiitd.edu.in/raghava/cppsite/) [122, 123]. CPP-protein conjugates were shown to enter cells either via direct translocation or via one of the endocytosis pathways after CPP binding to a cell-surface receptor. While the CPP-cargo uptake into endosomes could be highly efficient, its subsequent release to the cytosol is less effective, frequently leading to endosomal entrapment and degradation of the cargo protein [122, 123]. Yet, recent efforts by a number of groups proved that endosomal release could be achieved successfully by utilizing clever engineering strategies and better CPPs [124], thus signaling the shift in paradigm in protein drug development. Schepartz group for example, demonstrated that large enzymes such as SNAP-tag and Argininosuccinate Synthetase could be successfully delivered into cellular cytoplasm upon fusion to a Zn-finger domain that exposes a particular penta-arginine motif on the surface of an alpha-helix [125, 126].

Supercharging proteins is another method for protein delivery. In such a strategy, GFP with a highly positively charged surface could deliver cargo proteins by binding to the negatively charged membrane and internalizing through the endocytosis mechanism [127]. Furthermore, fusion of several highly positively charged human proteins to cargo proteins resulted in successful intracellular delivery both *in vitro* and *in vivo* [128]. Another interesting strategy for protein delivery is based on bacterial toxins such as anthrax lethal toxin [129, 130], pseudomonas aeruginosa Endotoxin A [131, 132], diphtheria toxin [133] and botulinum neurotoxin [134]. These toxins possess an intrinsic machinery to deliver bacterial proteins into the cytosol by binding to a particular cellular receptor through a receptor-binding domain and stimulating membrane translocation and endosomal release via another domain. The receptor-binding domain of bacterial toxins could be substituted to recognize a different receptor, thereby redirecting the uptake system to a receptor of choice [135]. Additional methods of protein delivery have been developed that rely on the coupling of the cargo protein to lipid or gold nanoparticles [136–138].

An alternative approach to protein delivery relies on intracellular delivery of mRNA that encodes the protein of interest. Once inside the cells, mRNA is translated by the cellular machinery, leading to the intracellular production of the desired protein. Since mRNA does not integrate into host genome and eventually degrades, protein expression remains transient, avoiding undesired long-term effects. Yet, just like proteins, negatively-charged mRNA does not cross the cellular membrane on its own and needs to be coupled to a cell-delivery component such as CPP, polymers, virus-like particles, or lipid nanoparticles (LPNs) [139, 140]. The remarkable success of the protein delivery through LPN-encapsulating mRNA has been demonstrated in the creation of vaccines for SARS-CoV2. Many other LPN-mRNA formulations have been developed and are undergoing clinical trials for prevention and treatment of infectious diseases, cancer, and genetic diseases [140]. One of key benefits of the mRNA-based platform is its ability to facilitate rapid production and modification of therapeutic proteins, which is especially important in fighting rapidly-mutating viruses. Yet, this strategy also has its limitations, including LNP endosomal entrapment and degradation and inability to target specific cells. Comparison of several protein delivery methods have been performed by the Plueckthun group and revealed that the efficiency of different methods varies considerably and depends on the type of cell lines used in the experiment [133].

Some of the above protein delivery approaches have been already explored for the delivery of Ras inhibitors. In an interesting approach, a human IgG1 antibody has been developed that internalizes into the cellular cytosol and selectively binds to Ras-GTP, inhibiting Ras downstream signaling. The internalization of the antibody here was achieved by introducing a variable light chain domain that promotes endocytosis by binding to the heparin sulfate proteoglycan that is expressed on the cell surface [141]. More importantly, this antibody undergoes conformational changes in response to the acidified pH of early endosomes, resulting in endosomal membrane pore formation and subsequent antibody escape to the cytosol. The Ras-targeting IgG1 was further engineered to introduce a tumor-associated integrin-binding RGD motif, facilitating successful delivery of the antibody into tumor tissues upon systemic administration and potent anti-tumor activity in a subset of Ras-mutated tumor xenografts in mice [120]. In another recent work, Nomura et al explored fusion of 51 different combinations of Ras binding domains to various CPPs [142]. The best candidates consisting a fusion of the Raf RBD engineered protein [39] and penetratin CPP could successfully reach the cytosol, competitively inhibit Ras/effector interactions and exert anticancer activity in several Ras-mutated cancer cell lines [142]. In another study, an optimized type III secretion system from Salmonella typhimurium was utilized to successfully deliver Ras-binding DARPins and monobodies into cellular cytoplasm and blocked functional signalling [143]. Furthermore, LNP-mRNA platform was used for efficient intracellular delivery of the Ras-binding iDab [144].

## CONCLUSIONS AND FUTURE CHALLENGES

In recent years a large number of Ras-binding proteins have been engineered from various scaffolds. Among such proteins, some recognize all Ras isotypes and oncogenic mutants while others are selective for a particular mutant or isotype. Various studies proved that interruption of Ras-effector interactions, Ras dimerization, Ras nucleotide exchange, all can lead to the desired anti-cancer effect. These engineered inhibitors are a powerful tool to study Ras biology and to determine sensitivity of various cancer cell lines to inhibition of Ras activity. In addition, interesting new strategies emerged beyond Ras inhibition that include stimulation of Ras-regulated tumor suppressor pathways and degradation of Ras oncogenic mutants. Engineered Ras binders could be used as competitors when screening for small molecules that bind to the same Ras binding interface with high affinity. In parallel, a number of promising approaches are emerging for intracellular protein delivery. These strategies should be utilized in future to examine the beneficial activity of Ras-binders and inhibitors and should further facilitate the development of protein-based Ras therapeutics.

## Abbreviations

CPP: cell-penetrating peptide
GAP: GTPase-activating proteins
GFP: green fluorescent protein
GEF: Guanine nucleotide Exchange Factors
GDP: Guanosine diphosphate
GTP: Guanosine triphosphate
IgG: Immunoglobulin G
YSD: yeast surface display
Proteolysis-targeting chimeras PROTAC: Proteolysis-targeting chimeras
RalGDS: Ral guanine nucleotide dissociation stimulator
RAS-Association RA: RAS-Association domain
RBD: Ras binding domain
IAC: intracellular antibody capture technique
CDRs: Complementary Determining Regions
Fabs: fragment antigen-biding
Fv: variable domains
or scFv: single chain variable domains
iDab: intracellular single variable domain antibody
ACE2: angiotensin-converting enzyme 2
PD: phage display
RASSF: Ras Association Domain Family
FN3: fibronectin type III domain
